# Systematic literature review assessing tobacco smoke exposure as a risk factor for serious respiratory syncytial virus disease among infants and young children

**DOI:** 10.1186/1471-2431-12-81

**Published:** 2012-06-21

**Authors:** Joseph R DiFranza, Anthony Masaquel, Amy M Barrett, Ann D Colosia, Parthiv J Mahadevia

**Affiliations:** 1 Department of Family Medicine and Community Health, University of Massachusetts Medical School, 55 Lake Avenue North, Worcester, MA, 01655, USA; 2 MedImmune, Health Outcomes & Pharmacoeconomics, One MedImmune Way, Gaithersburg, MD, 20878, USA; 3 RTI Health Solutions, 3040 Cornwallis Road, P.O. Box 12194, Research Triangle Park, NC, 27709-2194, USA

**Keywords:** Respiratory syncytial virus, Respiratory infection, Tobacco, Smoking, Children

## Abstract

**Background:**

The role of environmental tobacco smoke (ETS) exposure as a risk factor for serious respiratory syncytial virus (RSV) disease among infants and young children has not been clearly established. This systematic review was conducted to explore the association between ETS exposure and serious RSV disease in children younger than 5 years, including infants and young children with elevated risk for serious RSV disease.

**Methods:**

A systematic review of English-language studies using the PubMed and EMBASE databases (1990–2009) was performed to retrieve studies that evaluated ETS as a potential risk factor for serious RSV illness. Studies assessing risk factors associated with hospitalization, emergency department visit, or physician visit due to RSV (based on laboratory confirmation of RSV or clinical diagnosis of RSV) in children under the age of 5 years were included.

**Results:**

The literature search identified 30 relevant articles, categorized by laboratory confirmation of RSV infection (n = 14), clinical diagnosis of RSV disease (n = 8), and assessment of RSV disease severity (n = 8). Across these three categories of studies, at least 1 type of ETS exposure was associated with statistically significant increases in risk in multivariate or bivariate analysis, as follows: 12 of 14 studies on risk of hospitalization or ED visit for laboratory-confirmed RSV infection; 6 of 8 studies of RSV disease based on clinical diagnosis; and 5 of the 8 studies assessing severity of RSV as shown by hospitalization rates or degree of hypoxia. Also, 7 of the 30 studies focused on populations of premature infants, and the majority (5 studies) found a significant association between ETS exposure and RSV risk in the multivariate or bivariate analyses.

**Conclusion:**

We found ample evidence that ETS exposure places infants and young children at increased risk of hospitalization for RSV-attributable lower respiratory tract infection and increases the severity of illness among hospitalized children. Additional evidence is needed regarding the association of ETS exposure and outpatient RSV lower respiratory tract illness. Challenges and potential pitfalls of assessing ETS exposure in children are discussed.

## Background

Almost all children contract respiratory syncytial virus (RSV) by 2 years of age [1]. RSV causes upper airway infections, bronchiolitis, wheezy bronchitis, and pneumonia. Although most infections produce mild disease, RSV is a major cause of hospitalization in infants [2] and can be fatal [3]. Premature infants, infants with bronchopulmonary dysplasia (BPD) (chronic lung disease of prematurity), and infants with congenital heart disease are at risk for serious infections [4,5]. Risk factors for RSV infection include exclusive bottle feeding [6,7], having older brothers or sisters in the household [6,8,9], male gender [10], low birth weight [10,11], prematurity [6], household crowding [12], and young chronologic age [9]. Although exposure to environmental tobacco smoke (ETS) is a risk factor for asthma, wheezing, decreased pulmonary function, otitis media, cough, and lower respiratory tract infections (LRTIs) in general [13], its role in the development of serious RSV disease among infants and young children is less clear and has been a topic of interest among the healthcare community [14].

In this systematic review, we evaluated the evidence of an association between ETS exposure and serious RSV disease among children younger than 5 years. To obtain the broadest understanding of whether ETS exposure affects the risk of serious RSV disease, we placed no limits on the type of proxy measures of ETS exposure. Understanding the evidence basis for ETS exposure and RSV could highlight the need to direct healthcare resources or intervention programs toward this potentially modifiable risk factor.

## Methods

### Patient population

In this systematic literature review, we included studies of infants and children up to 5 years of age, including studies of children at high risk for serious RSV disease. We defined the high-risk population as patients with prematurity, BPD, or congenital heart disease. All other patient populations were defined as general populations, which consisted predominantly of children not defined as high risk.

### Search strategy

Following PRISMA guidelines, we conducted a systematic review of the association of ETS exposure with serious RSV disease by searching broadly for studies assessing various risk factors, including ETS exposure, for RSV, bronchiolitis, or LRTI caused by RSV or bronchiolitis.[15] We searched the PubMed (including MEDLINE) and EMBASE databases for English-language studies published and indexed between 1990 and April 2009. No additional relevant unpublished studies were obtained. Studies that identified ETS exposure as a risk factor might be more likely to mention ETS in the title or abstract than studies that evaluated ETS exposure in a set of other risk factors, but did not find it to be significantly associated with increased risk. To minimize article selection bias across studies, we searched for articles that assessed any risk factors for RSV as well as bronchiolitis, because 50% to 80% of winter bronchiolitis is due to RSV in infants and young children [1]. For PubMed, the National Library of Medicine Medical Subject Headings (MeSH terms) used in the search included *tobacco smoke pollution/adverse effectsrespiratory syncytial virus infectionsbronchiolitis viral*, and *respiratory tract infections* in combination with the MeSH subheadings *epidemiologyetiology*, and *complications*. The MeSH heading *respiratory tract infections* was combined with the keywords *syncytial* or *RSV* OR *bronchiolitis* or the MESH term *tobacco smoke pollution/adverse effects*. Studies that included risk assessment were identified using the following MeSH headings: *analysis of variance* (which includes multivariate analysis), *probability* (which includes proportional hazards model, odds ratio, risk, risk assessment, and risk factors); and *case–control studies* or *cohort studies*, which were combined with the terms *relative risk* OR *hazard ratio* OR *odds ratio*. The search identified studies with the following combination of these terms: (1) any disease-related term plus any risk assessment term; or (2) any disease-related term plus the smoke exposure term. We did not search for unpublished studies or reports.

### Study selection

**Figure 1:**
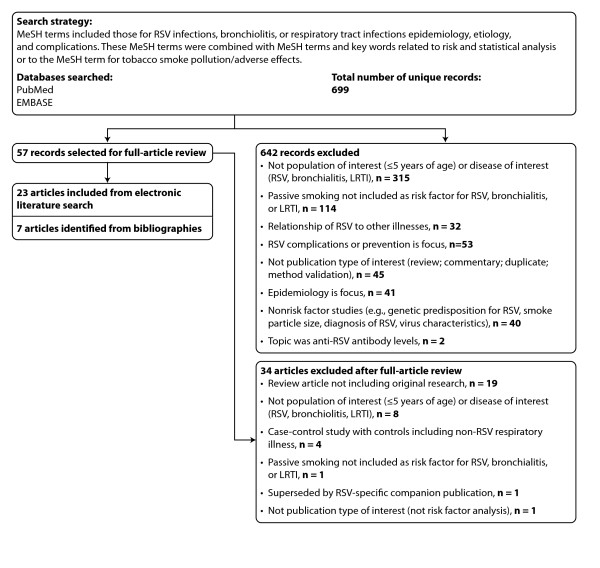
**PRISMA Flow Diagram: Summary of Exclusions and Rationales for Exclusion During Phases of Systematic Literature Review.** PRISMA = Preferred Reporting Items for Systematic Reviews and Meta-Analyses; LRTI = lower respiratory tract infection; MeSH = medical subject headings; RSV = respiratory syncytial virus.

Each relevant article was read by several authors and the bibliographies of included articles were reviewed for additional studies. We excluded studies that did not assess disease risk or did not include ETS exposure as a risk factor. Figure 1 presents a summary of exclusions and rationales for exclusion during successive rounds of review.

### Data extraction

Study details were extracted into tables, and table content was verified by a second author and by a reviewer not involved in the data extraction. The authors discussed each article to reach consensus regarding the study details. For each study the following data were extracted: reference, publication year, country origin, study design, study population size and description, assessment method for ETS exposure (e.g., maternal smoking through birth certificate data), method of ascertainment of RSV status (if any, such as through laboratory confirmation or clinical diagnosis), disease outcome evaluated (e.g., hospitalization for RSV), and results and significance of multivariate, bivariate, and other statistical analyses. Outcomes related to the presence of RSV antibodies without evidence of hospitalization, ED visit, or physician visit related to RSV were not extracted. The principal summary measures were adjusted odds ratios (aORs), adjusted rate ratios (aRRs), or adjusted hazard ratios (aHRs) for multivariate analyses and odds ratios (ORs) for bivariate analyses. Several studies that did not perform multivariate analyses performed between-group comparisons and presented *P* values.

### Assessment of risk of bias

Among the studies meeting the inclusion criteria, we evaluated the risk of bias at the outcome and study level using the Cochrane risk of bias tool [16]. The Cochrane risk of bias tool was developed primarily for use in interventional studies; we used the tool to examine factors specific to sources of bias frequently found in observational studies. The studies were examined for risk of bias that would potentially influence the association of ETS exposure with serious RSV disease. The studies were judged regarding evidence of misclassification bias of RSV disease, selective reporting bias, confounding bias, exposure ascertainment bias, or participant selection bias. Disease misclassification bias was assessed based on the likelihood of the study population having an LRTI that was not due to RSV. We expect that disease misclassification would underestimate the association between ETS exposure and serious RSV disease. Selective reporting bias was evaluated based on whether outcomes related to risk factors were clearly reported. Confounding bias was assessed based on whether the study controlled for the effects of other variables through multivariate analysis. We expect that unadjusted results may overestimate the association between ETS exposure and serious RSV disease. Exposure ascertainment bias was assessed based on whether the ETS exposure variables were described clearly and determined by the study authors to be adequate measures of ETS exposure in the study population. Finally, participant selection bias was pointed out in case–control studies in which the control group consisted of ill patients with non-RSV respiratory disease, and therefore, the effect of ETS exposure on serious RSV disease could be biased toward the null.

## Results

The search of PubMed and embase databases yielded 699 unique results, and the abstracts were reviewed for relevance (Figure 1). Of these, 676 were excluded through successive rounds of review, the majority because the studies did not focus on a disease of interest (RSV, bronchiolitis, or LRTI attributable to RSV or bronchiolitis), were not conducted in a population of interest (children younger than 5 years without serious nonrespiratory disease, such as cancer or organ transplant), or did not assess ETS exposure.

Full review of 57 articles resulted in the exclusion of 34 articles, leaving 23 articles identified by the electronic search. The addition of 7 articles identified from references cited (4 were published before 1990) produced a total of 30 relevant articles.

### Overview of studies

The results are organized by study characteristics and include a bias assessment summary for each study. (The full bias assessment is presented in Additional file 1: Table S1) Table 1 presents 14 studies that established a diagnosis of RSV disease by confirmatory laboratory testing. These studies compared children with RSV infections with children without respiratory illness and assessed whether ETS exposure increased the risk for developing a serious RSV infection. Table 2 presents 8 studies that did not confirm suspected cases of RSV disease with laboratory testing, but relied on the clinical diagnosis of RSV or bronchiolitis, often from medical charts or insurance data. These studies also compared children with RSV or bronchiolitis infections with children without respiratory illness. Table 3 presents 8 studies that examined the impact of ETS exposure on the severity of RSV disease as measured by hospitalization or degree of hypoxia. These studies started with a sample of children infected with RSV and assessed whether ETS exposure increased the severity of the RSV infection. Within each group of studies, we considered studies of premature infants separately because this population is at higher risk for serious RSV disease than the general population of children [17,18]. We also present the results by study design (e.g., cohort, case–control) in each table.

**Table 1:** Studies Reporting Risk of Developing Serious RSV (Laboratory-Confirmed RSV; N = 14)

**Study, Year, Country**	**Design and Population**	**ETS Exposure**	**Outcome**	**Results**	**Bias Assessment/Comment**
*Prospective cohort studies in premature infants*					
Broughton 2005 United Kingdom [19]	Prospective study of 126 premature infants (GA <32 wks; 40% developed BPD)	Maternal smoking during pregnancy	RSV LRTI (41% hospitalized)	aOR, 4.85 (95% CI, 1.61–14.58); *P* = 0.005	No significant bias concerns affecting the relationship of ETS and outcome
		Parental smoking in home	RSV LRTI	NS aOR, 0.81 (95% CI, 0.19–3.37); *P* = 0.771	
		Maternal smoking during pregnancy	Hospital admission (all cause; 56% of admissions were RSV LRTI)	NS aOR, 1.19 (95% CI, 0.20–7.07); *P* = 0.849	
		Parental smoking in home	Hospital admission	aOR, 3.39 (95% CI, 1.08–10.63); *P* = 0.003	
		Maternal smoking during pregnancy	Length of hospital stay	NS, *P* = 0.150 (OR not reported)	
		Parental smoking in home	Length of hospital stay	*P* < 0.001 (OR not reported)	
Carbonell-Estrany 2001 Spain [9]	Prospective, longitudinal study of 999 premature infants (GA ≤32 wks)	Days of smoke exposure	RSV hospitalization	aOR, 1.63 (1.05–2.56); *P* = 0.031	No significant bias concerns affecting the relationship of ETS and outcome
Figueras-Aloy 2008 Spain [20]	2-cohort study of premature infants (GA 32–35 wks); 202 cases hospitalized for RSV and 5239 controls not hospitalized for respiratory illness	Maternal smoking during pregnancy	RSV hospitalization	aOR, 1.61 (95% CI, 1.16–2.25); *P* = 0.004	Authors note relatively high loss to follow-up of 12% of children fulfilling inclusion criteria. Both ETS exposure variables were significant in bivariate analysis at *P* < 0.01, but when included in multivariate model, only prenatal smoking was significant, possibly due to misclassification of ETS exposure
		≤2 smokers in home	RSV hospitalization	NS in multivariate model	
				Significant in bivariate analysis, OR 1.59 (95% CI, 1.12–2.26); *P* = 0.01	
Law 2004 Canada [10]	Prospective cohort study of 1832 premature infants (GA 33–35 wks)	≥2 smokers in household	RSV hospitalization	aOR, 1.87 (95% CI, 1.07–3.26); *P* = 0.027	No significant bias concerns affecting the relationship of ETS and outcome
*Case–control study in premature infants*					
Figueras-Aloy 2004 Spain [21]	Case–control study of premature infants (GA 33–35 wks); 186 cases hospitalized for RSV; 371 controls born at same time as cases	Maternal smoking during pregnancy	RSV hospitalization	NS in multivariate model	No significant bias concerns affecting the relationship of ETS and outcome
				Significant in bivariate analysis OR, 1.62 (95% CI, 1.08–2.42); *P* = 0.027	
		Maternal smoking at home	RSV hospitalization	NS in bivariate model	
				OR, 1.49 (95% CI, 1.01–2.18); *P* = 0.055	
		≥2 smokers at home	RSV hospitalization	NS in bivariate model	
				OR, 1.41 (95% CI, 0.92–2.14); *P* = 0.146	
*Prospective cohort studies in the general population*					
Holberg 1991 US [22]	Prospective birth cohort study of 1179 healthy infants followed for 1 year	Maternal smoking	RSV diagnosed in an office visit	NS in multivariate model	No significant bias concerns affecting the relationship of ETS and outcome
				Rate ratio, 1.0 (95% CI, 0.3–3.5)	
von Linstow 2008 Denmark [6]	Prospective birth cohort study of 217 children followed for 1 year	Smoking in household	RSV hospitalization	aOR, 5.06 (95% CI, 1.36–18.76); *P* < 0.02	No significant bias concerns affecting the relationship of ETS and outcome; to reduce problems with colinearity, only 1–2 variables from each group of covariates (e.g., social variables, smoking parameters) were included in the multivariate model.
		Maternal smoking during pregnancy	RSV hospitalization	NS in multivariate model (OR not reported)	
				Significant in univariate model	
				OR, 4.19 (95% CI, 1.21–14.53); *P* = 0.024	
*Case–control studies in the general population*					
Bulkow 2002 US [12]	Case–control study of Alaska native children aged <3 years with 204 cases and 338 controls	Smoker in household	RSV hospitalization	NS in multivariate model	Unclear risk of ETS exposure misclassification because of high prevalence of smoking and frequency of indoor visiting among households during winter RSV season; low risk of other types
				Significant in bivariate analysis, OR, 1.61; *P* ≤ 0.018	
Gurkan 2000 Turkey [23]	Case–control study of 28 cases and 30 controls aged 2–18 months	▪Nonsmoking parents ▪Only smoker mother ▪Only smoker father ▪Both parents smokers	RSV bronchiolitis admitted to the ED Serum cotinine assessed during ED visit and 1 month later	Significant differences in cases vs. controls (*P* < 0.05) for all ETS exposure variables; however, only father smoker was more prevalent in the control than case group	No multivariate analysis performed (confounding bias)
				Significant differences in cases vs. controls (*P* < 0.05) in cotinine levels for both parents smokers vs. both parents nonsmokers and for only mother smoker vs. both parents nonsmokers in the control group	
Hall 1984 US [24]	Case–control study of 29 cases and 58 controls hospitalized with non respiratory acute illness	Smoking in household	RSV hospitalization	Significant difference in smoking in household in cases (76%) vs. controls (40%) (*P* < 0.05)	No multivariate analysis performed (confounding bias)
Hayes 1989 American Samoa [25]	Case–control study of children aged <1 year (20 cases and 15 well controls)	Smoker in household	RSV hospitalization (53% laboratory-confirmed)	Significant difference in smoker in household in cases (92%) vs. well controls (53%) (*P* = 0.04)	No multivariate analysis performed (confounding bias)53% of hospitalizations were laboratory-confirmed RSV
Nielsen 2003 Denmark [5]	Case–control study of 1252 cases in children aged <2 years and 5 controls for each case	Maternal smoking during pregnancy from the Medical Birth Register	RSV hospitalization	aOR, 1.56 (95% CI, 1.32–1.98)	No significant bias concerns affecting the relationship of ETS and outcome
Reeve 2006 Australia [11]	Case–control study with 271 cases and 542 controls (median age 6 mo)	Maternal smoking	RSV hospitalization	NS in main multivariate modelBivariate OR not reported CART analysis performed to define groups that are most homogeneous with regard to the outcome of RSV hospitalization. CART analysis found that smoking was a risk factor in children with birthweight >2500 g and single mothers (41.0% hospitalized vs. 26.9% for single nonsmoking mothers)Smoking was not significant for any other group	Analysis was weakened by reliance on a questionnaire that did not seek to quantify ETS exposure and by the absence of laboratory confirmation of ETS exposure.63 participants were excluded due to data unavailability (37 of these were missing the mother’s smoking status and 47 had proven RSV), although the missing data were not statistically significant
Stensballe 2006 Denmark [26]	Case–control study of 2564 cases and 12 816 controls from birth to 18 months	Any maternal smoking during pregnancy and lactation	RSV hospitalization	aOR, 1.35 (95% CI, 1.20–1.52); *P* < 0.001	No significant bias concerns affecting the relationship of ETS and outcome

aOR = adjusted (multivariate) odds ratio; BPD = bronchopulmonary dysplasia (now chronic lung disease); CART = Classification and regression tree; CI = confidence interval; ED = emergency department; ETS = environmental tobacco smoke; OR = odds ratio; GA = gestational age; LRTI = lower respiratory tract infection; NS = not significant; RSV = respiratory syncytial virus.

**Table 2:** Studies reporting risk of developing serious rsv (clinical diagnosis of rsv illness; N = 8)

**Study, Year, Country**	**Design and Population**	**Smoke Exposure**	**Outcome**	**Results**	**Bias Assessment**
*Cohort study in premature infants*					
Gavin 2007 US [27]	Retrospective cohort study of 2098 premature infants (GA 32–35 weeks) in the Texas Medicaid program	Maternal smoking status during pregnancy from the birth certificate	Insurance claims for bronchiolitis or RSV hospitalization in the first year of life	NS, aOR, 0.78 (95% CI, 0.38–1.61)	Clinical diagnosis of RSV leading to misclassification could underestimate ETS exposure risk
*Cohort studies in the general population*					
Boyce 2000 US [4]	Retrospective cohort study of children aged <3 years in the Tennessee Medicaid program from 1989–1993, with 248 652 child-years of follow-up	Maternal smoking status during pregnancy from the birth certificate	Insurance claims for bronchiolitis or RSV hospitalization in the first year of life	aRR, 1.3 (95% CI, 1.2–1.4)	Clinical diagnosis of RSV leading to misclassification could underestimate ETS exposure risk
Carroll 2007 US [28]	Retrospective cohort study of 101 245 term infants enrolled in the Tennessee Medicaid program	Maternal smoking status during pregnancy from the birth certificate	Insurance claims for bronchiolitis or RSV pneumonia in the first year of life	▪Hospitalization aOR, 1.28 (95% CI, 1.20–1.36)	Clinical diagnosis of RSV leading to misclassification could underestimate ETS exposure risk
				▪ED visit aOR, 1.22 (95% CI, 1.13–1.31)	
				▪Clinic visit aOR, 1.06 (95% CI, 1.01–1.12)	
				▪Bronchiolitis diagnosis aHR, 1.14 (95% CI, 1.10–1.18)	
Koehoorn 2008 Canada [29]	Retrospective cohort study of 93 058 infants aged 2–12 months	Maternal smoking status during pregnancy from perinatal database	Diagnostic codes for bronchiolitis for outpatient visits or hospitalizations	▪Inpatient onlyaHR, 1.47 (95% CI, 1.27–1.69)	Clinical diagnosis of bronchiolitis leading to misclassification could underestimate ETS exposure risk
				▪Outpatient or inpatient NS in multivariate model, aHR, 1.03 (95% CI, 0.97–1.09)	Maternal smoking during pregnancy was significant in bivariate analysis for both case definitions, but when included in the multivariate models, it was significantly associated only with the inpatient (more severe) case definition
				▪Significant in bivariate analysis, OR 1.14 (95% CI, 1.08–1.21); no *P* value reported	
Marbury 1996 US [30]	Prospective cohort study of 1424 children with private insurance followed to age 2 years	Maternal smoking status	Diagnosis of bronchiolitis from electronic medical records	NS, aRR, 1.3 (95% CI, 0.8–2.2); no *P* value reported	Clinical diagnosis of bronchiolitis leading to misclassification could underestimate ETS exposure risk The authors noted that smokers were less likely to participate in the study (the Indoor Air and Children’s Health Study) and that smokers who participate may differ from those who do not. They also noted the possibility of underreporting of smoking
Reese et al., 1992 Australia [18]	Retrospective cohort study of 491 patients up to age 17 years admitted to an Australian children’s hospital June-Dec. 1987 for whom urinary cotinine levels were available.	Urinary cotinine level, analyzed without knowledge of exposure status or diagnosis	Hospitalization with diagnosis of bronchiolitis vs. a non-respiratory diagnosis (limited to patients aged 5–15 mos in the nonrespiratory illness group)	Elevated cotinine levels found in bronchiolitis vs. nonrespiratory illness group (*P* < 0.02) Subanalysis of the bronchiolitis group by RSV status found no significant difference between RSV-positive (n = 16) and RSV-negative (n = 23) patients; both subgroups had elevated cotinine vs. the nonrespiratory illness group	Risk of confounding not clear; regression analysis appears to have been performed but was insufficiently reported (no aORs reported)
	Among those with cotinine levels, 41 patients (aged 5–15 mos.) were diagnosed with bronchiolitis				
*Case–control studies in the general population*					
Holman 2003 US [3]	Case–control study of 224 infants aged <1 year who died from bronchiolitis, and 2336 controls	Maternal smoking status during pregnancy	Bronchiolitis death from death certificate data	aOR, 1.6(95% CI, 1.0–2.6)	Clinical diagnosis of bronchiolitis from death certificate leading to misclassification could underestimate ETS exposure risk
McConnochie 1986 US [31]	Case–control study of 53 cases of bronchiolitis and 106 controls in children aged <2 years presenting to a physician’s officeBivariate analysis included 3 ETS exposure variables, but only “passive smoking” was included in the multivariate analysis	Any passive smoking	Bronchiolitis from diagnostic registry and record review	aOR, 3.87 if no family history of asthma (no CI or *P* value reported)aOR, 4.03 if family history of asthma (no CI or *P* value reported)	Clinical diagnosis of bronchiolitis leading to misclassification could underestimate ETS exposure risk Interviews related to smoking status were conducted approximately 7.8 years after the bronchiolitis episodes; current and former smokers at the time of the interview were assumed to be smoking at the time of the bronchiolitis episode
		Smoking in household	Bronchiolitis	Bivariate OR, 3.21 (95% CI, 1.42–7.25)	
		Mother smokes	Bronchiolitis	Bivariate OR, 2.33 (95% CI, 1.19–4.57)	
		Father smokes	Bronchiolitis	NS in bivariate model, OR 1.71 (95% CI, 0.87–3.33)	

aHR = adjusted hazard ratio; aOR = adjusted (multivariate) odds ratio; aRR = adjusted rate ratio; CI = confidence interval; ED = emergency department; ETS = environmental tobacco smoke; GA = gestational age; LRTI = lower respiratory tract infection; NS = not significant; RSV = respiratory syncytial virus; OR=odds ratio.

**Table 3:** Studies examining disease severity (N = 8)

**Study, Year, Country**	**Design and Population**	**Smoke Exposure**	**Outcome**	**Results**	**Bias Assessment**
*Risk of hospitalization among premature children with RSV illness*					
Groothuis et al., 1988 US [32]	Prospective cohort study of 30 premature infants aged <2 years with BPD receiving home oxygen therapy; participants followed for 5 mos (Dec-Apr)	Smokers in home	Risk of hospitalization (11 of 16 with RSV hospitalized) vs. outpatient treatment	NS; bivariate analysis reported and *P* value not given	No multivariate analysis performed (confounding bias)
*Risk of hospitalization among children in the general population with RSV illness*					
Al-Shehri 2005 Saudi Arabia [33]	Case–control study; 51 children aged ≤5 years hospitalized for bronchiolitis (cases) and 115 children with bronchiolitis but not hospitalized (controls); 40% of cases were RSV	History of exposure to smoking	Risk of hospitalization vs. outpatient treatment for bronchiolitis	aOR, 2.51 (95% CI, 2.11–3.73); *P* = 0.05	Risk of participant selection bias because both cases and controls had bronchiolitis
Hall 2009 US [2]	919 children aged <5 years with laboratory-confirmed RSV infections	Smoking in household	Risk of hospitalization vs. outpatient treatment	NS in multivariate analysis (no aOR, CI, or P value)NS in bivariate analysis (*P* = 0.43)	No significant bias concerns affecting the relationship of ETS and outcome
		Mother smokes	Risk of hospitalization vs. outpatient treatment	Not included in multivariate analysis NS in bivariate analysis *(P* = 0.21)	
Somech 2006 Canada [34]	Prospective study of 195 infants (mean age 3.8 months) with laboratory-confirmed RSV infection	Exposure to smoke from at least one family member	Hospitalization (113) vs. outpatient treatment (82) of RSV	ETS exposure was unrelated to hospitalization (*P* value not reported)	No multivariate analysis performed (confounding bias)
*Disease severity in children in the general population hospitalized with RSV illness*					
Al-Sonboli 2006 Yemen [35]	Prospective study of 325 children aged ≤2 with acute respiratory illness seeking emergency or outpatient services at a hospital (82% RSV)	Smoking in household	Severe hypoxia among RSV-positive group	aOR, 3.8 (95% CI, 1.5–9.8); *P* = 0.002	No description of how smoke exposure or other family characteristics were ascertained (exposure bias)
Bradley 2005 US [36]	Prospective evaluation of 206 infants hospitalized with their first episode of severe RSV bronchiolitis	Current maternal smokingMaternal smoking status during pregnancy	Lowest oxygen saturation rate	Current maternal smoking was associated with lower oxygen saturation, *P* =0.05No effect of smoking during pregnancy only (n = 10)	No significant bias concerns affecting the relationship of ETS and outcome
Chatzimichael 2007 Greece [37]	Prospective study of 240 children aged 6–24 months hospitalized for bronchiolitis	Exposure to >5 cigarettes per day in the home; children with prenatal exposure were excluded	Disease severity measured with a clinical rating tool that included hypoxemia	aOR, 2.2 (95% CI, 1.1–3.6); *P* = 0.003	Unclear RSV disease classification; severity tool used
Sritippayawan 2006 Thailand [38]	Study of 19 children (median age 9 months) admitted to the hospital with laboratory-confirmed RSV LRTI	Exposure measured by urinary cotinine	Hypoxemia (oxygen saturation <92%)	Cotinine was detected in 100% of infants with hypoxia vs. 33% of those without hypoxia; *P* = 0.01	High risk of selective reporting biasRisk of confounding not clear; regression analysis appears to have been performed but was insufficiently reported so it was not possible to tell which factors were controlled for

aOR = adjusted (multivariate) odds ratio; BPD = bronchopulmonary dysplasia; CI = confidence interval; ETS = environmental tobacco smoke; LRTI = lower respiratory tract infection; NS = not significant; RSV = respiratory syncytial virus.

The methods used to assess ETS exposure status varied widely and included mother’s prenatal smoking status, mother’s postnatal smoking status, father’s smoking status, smoking in the home, number of smokers in the household, number of cigarettes smoked in the home, more than five cigarettes smoked per day in the home, days of smoke exposure, history of exposure to smoking, smoking by daycare provider, and cotinine levels.

Some of the studies summarized included more than one source of ETS exposure, but no study detailed missing ETS exposure status data. In the studies reporting multiple ETS exposure, we have presented all results based on the different sources (e.g., maternal smoking during pregnancy and smokers in the household) because it is difficult to determine in each setting which of several proxies for total ETS exposure results in the least misclassification error. Few of the multivariate studies provided a rationale for their selection of control variables. We therefore included positive bivariate results in our tables when bivariate and multivariate analyses produced disparate conclusions.

### ETS exposure and laboratory-confirmed RSV disease

The studies in Table 1 seek to answer the question, does ETS exposure increase the risk that an uninfected child will develop serious RSV? In 14 studies, the diagnosis of RSV infection was confirmed by laboratory testing (Table 1). Of these, 12 studies showed a significant adverse impact of ETS exposure on serious RSV in bivariate or multivariate analysis as measured by at least one exposure variable. These studies examined the association of ETS exposure and other factors on the risk of ED visit [23] or hospital admission [5,6,9,10,12,19-21,24-26] for laboratory-confirmed RSV LRTI. Two studies examining laboratory-confirmed RSV did not find a statistically significant association between ETS exposure and serious RSV: a cohort study of outpatients in the United States assessing the risk of a child presenting to the pediatrician’s office with an RSV infection [22], and an Australian case–control study assessing the risk of RSV hospitalization [11].

Four of these 14 studies contained at least one significant association, but found mixed results with different ETS exposure variables [6,19,20,23]. One prospective cohort study in premature infants found that maternal smoking and parental smoking in the home had conflicting associations with three different outcomes: RSV LRTI, all-cause hospital admission, and length of hospital stay [19]. Measures of smoke exposure in the home and maternal smoking gave conflicting results when evaluated in 2 studies [6,20], including a study in premature infants [20]. Finally, a small case–control study in a general population in Turkey found that several exposure variables (only mother smoking, both parents smoking, any parent smoking, and serum cotinine) were associated with significant increase in risk of admittance to the ED; however, only father smoking was significantly associated with a decrease in risk [23].

Of the studies of laboratory-confirmed RSV hospitalization described in Table 1, 5 were conducted in premature infants and 9 were conducted in general populations. All 5 studies of laboratory-confirmed RSV in premature infants found increased risk related to ETS exposure in either bivariate or multivariate analyses [9,10,19-21] (Table 1). The 2004 case–control study of infants with a gestational age of 33 to 35 weeks by Figueras-Aloy et al. [21] found an OR of 1.62 in the bivariate analysis that did not remain significant in the multivariate analysis. However, the same researchers published a much larger cohort study in 2008, in a premature population with a gestational age of 32 to 35 weeks, reporting a nearly identical aOR (1.61) for maternal smoking that remained significant in the multivariate analysis [20]. The authors of the 2004 study note that the prevalence of ETS exposure decreased in Spain during the years before the study (52% during the 1999–2000 RSV season compared with 30% in this study) because of anti-smoking campaigns or health education [21]. The significant association in the larger study suggests that the earlier study was underpowered for the multivariate analysis.

Of the 9 studies of laboratory-confirmed RSV illness in general populations described in Tables 1,2 were prospective cohort studies and 7 were case–control studies (Table 1). Risk of RSV LRTI in an outpatient office setting [22] and risk of RSV hospitalization from ETS exposure (smoking in the household, but not from maternal smoking during pregnancy) [6] were not significant in the cohort studies.

The 7 case–control studies of laboratory confirmed RSV illness in general populations ranged in size from 20 to 2,564 cases (Table 1), and all found a significant association between ETS exposure and risk of RSV hospitalization in either the multivariate or bivariate analyses, with the ORs from the 4 largest studies clustering tightly between 1.35 and 1.6. The 3 smallest case–control studies included in Table 1 did not provide ORs but compared ETS exposure prevalence between the cases and healthy controls [23-25]. All 3 found significant differences in ETS exposure in cases versus controls. The Turkish study also demonstrated significantly higher cotinine levels in serum samples from cases than from healthy controls [23]. In a case–control study of Alaska native children, risk was apparent in the bivariate analysis, but not in the multivariate analyses [12] (Table 1). The authors noted that misclassification of exposure status may have been a problem. The study was conducted in Alaska, where children spend much time indoors during the winter RSV season and could be exposed to ETS when visiting other houses or community buildings. This appears likely, because there were smokers living in the households of 59% of the controls and 68% of the cases. The consistency of findings associating ETS exposure and increased risk of serious RSV, coupled with the inconsistency of the variables associated with these significant results, highlights the challenges of assessing and categorizing ETS exposure.

### ETS exposure and clinically diagnosed RSV disease

As with the studies in Table 1, the studies in Table 2 seek to answer the question, does ETS exposure increase the risk that an uninfected child will develop serious RSV? In these studies, however, the RSV diagnosis was based on clinical findings rather than a laboratory test. Table 2 presents 8 studies, 6 of which reported a significant association between ETS exposure and bronchiolitis hospitalization or outpatient treatment. Because other organisms may cause bronchiolitis, there may be possible misclassification of RSV disease status [33].

The large general population cohort studies including bronchiolitis hospitalization as an outcome by Boyce et al. [4], Carroll et al. [28], and Koehoorn et al. [29] are in agreement with the 12 studies in Table 1 that identified an increased risk of RSV hospitalization associated with ETS exposure. Carroll and colleagues also demonstrated increased risk of ED visit or clinic visit for bronchiolitis and bronchiolitis diagnosis related to ETS exposure [28].

In other studies assessing risk of bronchiolitis LRTI in children presenting in the office setting, 2 studies found an increased risk for at least one ETS variable [28,31], whereas 2 other studies did not [29,30]. Of these, the positive study by Carroll et al. [28] had the greatest statistical power.

Only the Holman study assessed risk of death from bronchiolitis; the risk was increased by maternal smoking (aOR, 1.6) [3].

### ETS exposure and severity of RSV illness

The 8 studies in Table 3 address the question, given that a child has contracted RSV, does ETS exposure increase the severity of illness? Four studies compared ETS exposure in children hospitalized for RSV and children with RSV who were not hospitalized. Three of these studies, including a small study of premature infants with BPD, found no effect of ETS exposure on the likelihood of hospitalization for RSV [2,32,34]. One small study found an effect of ETS exposure, but only 40% of the hospitalized children were found to have RSV infections, with the remaining 60% representing bronchiolitis caused by other viruses [33].

Four studies examined clinical severity (degree of hypoxemia) of RSV LRTIs among children hospitalized for treatment. All 4 found that ETS exposure was associated with more severe illness (Table 3). Bradley et al. found that hypoxemia was associated with postnatal but not prenatal smoke exposure [36]. Chatzimichael et al. found that breastfeeding was protective against the effect of ETS exposure on disease severity [37]. Based on a small number of studies of RSV severity, ETS exposure does not appear to increase the risk of hospitalization versus outpatient treatment among children infected with RSV, but hospitalized RSV patients exposed to ETS have more severe disease.

### Summary of potential bias in observational studies of ETS exposure and serious RSV disease

As the studies reviewed were observational, we evaluated the primary sources of bias that could potentially affect the estimates of association with ETS exposure (Additional file 2). We found that 21 of the 30 studies confirmed the RSV diagnosis through laboratory testing for RSV or multiple respiratory illnesses including RSV in all of the study populations [2,5,6,9-12,19-26,32-36,38]. The remaining studies had a risk of disease misclassification bias of RSV because diagnostic codes (primarily for bronchiolitis) or diagnoses from medical records were used [3,4,27-31,37,39]. Misclassification of RSV disease may attenuate the association of ETS exposure and severe RSV disease, and the studies of clinically diagnosed RSV patients may provide conservative estimates of ETS exposure risk.

Overall, we identified few studies that had potential selective reporting bias, confounding bias, exposure ascertainment bias for ETS, or participant selection bias. Selective outcome reporting was a potential problem in 2 studies [38,39], meaning that outcomes were not described sufficiently to determine which factors were included and potentially controlled for in the analysis. Although confounding is a threat across observational studies (because it is difficult to control for both measureable and unmeasurable factors), multivariate analyses were not conducted in a number of studies [23-25,32,34,38], which could lead to an overestimate of the effect of ETS exposure. One study had a high risk of participant selection bias, where both cases and controls were diagnosed with bronchiolitis [33]. Only 2 studies had an unclear risk of ETS exposure [12,35]. Al-Sonboli and colleagues [35] did not describe how the ETS exposure data or other demographic data were obtained. In the Bulkow et al study, ETS exposure was highly prevalent in the community (in both cases and controls) and indoor visiting was frequent during the winter RSV season [12]; therefore, the ETS exposure variable “smoker in household” was not an adequate proxy for exposure in this population.

No statistical analysis of the risk of bias across studies was performed for this review.

## Discussion

The impact of ETS exposure on RSV disease in infants and young children is consistent among studies using laboratory confirmation of RSV infection and clinical diagnosis of bronchiolitis or RSV. Among 14 population-based studies that examined the risk of admission to the hospital or ED for RSV disease, 12 showed that at least one type of ETS exposure in each study was associated with a significant adverse outcome in the bivariate or multivariate analysis. The observation that ETS exposure increases the risk that a child will develop RSV disease that will require hospitalization is robust because these studies used different methods (prospective, retrospective, cohort, case–control) in different patient populations (infants who were premature, term, or with compromising conditions), in a variety of countries and cultures. In addition, the evidence suggests that ETS exposure is associated with more severe hypoxia among children hospitalized for RSV [35-38], and one study found an increased risk of mortality from bronchiolitis [3].

Among studies in premature infants, 5 [9,10,19-21] of 7 [9,10,19-21,27,32] found ETS exposure to be a significant risk factor in bivariate or multivariate analysis, including 5 of the 6 [9,10,19-21,32] studies assessing laboratory-confirmed RSV. One study contradicting this conclusion did not have laboratory confirmation of the diagnosis and relied on a claims database, rather than direct data collection, for ascertainment of the ETS exposure status [27]. Misclassification of disease status or missed diagnosis may have contributed to the negative findings in this study. The other was a small study of 30 premature infants with BPD on home oxygen therapy, 16 of whom developed RSV [32].

The evidence concerning whether ETS exposure increases the risk of mild RSV infection is much less convincing. In a very large study, Carroll et al. found ETS exposure to be associated with only a small increased risk of RSV illness (OR, 1.06) presenting in the outpatient setting [28]. If this OR represents the true risk, the Holberg [22] and Marbury [30] studies, which did not find a risk, would have been underpowered to detect it because of smaller sample sizes. Because nearly all children contract RSV during the first few years of life [1] and most cases are mild [2], there may be a ceiling effect. If children with no ETS exposure are almost certain to contract RSV, ETS exposure can increase the risk of mild infection only slightly.

Misclassification of ETS exposure is a major challenge in studying associations of ETS exposure with disease. Misclassification of exposure status produces a bias in the direction of reducing the apparent magnitude of the risk, leading to either an underestimate of the true ETS exposure risk or to a null finding. Misclassification of an infant or child’s ETS exposure comes from researchers’ use of one or a few measures of possible exposure, which actually can come through many avenues. Examples include *in utero* exposure through active and passive maternal smoking and postnatal smoking by the mother, father, other individuals living in the home, visitors, and babysitters. ETS exposure outside the home occurs in public places, day care settings, and houses of friends and relatives. No study in this review gathered exposure data for all potential sources. The smoking status of, for example, the parent is a poor proxy for this global exposure as demonstrated by cotinine level studies. Urinary or serum cotinine (a metabolite of nicotine) is an objective measure of ETS exposure but provides information regarding only the previous 48 to 72 hours of exposure [40]. In one study, infants whose parents reported that they did not smoke had mean cotinine levels that were 80% as high as those for infants with one smoking parent [39]. Some infants of nonsmoking parents had higher cotinine levels than some infants with two smoking parents [39].

Confounding bias is another potential obstacle to determining the association between ETS exposure and serious RSV disease. A few studies found an ETS exposure effect in bivariate analyses but not in multivariate analyses after adjustment for other factors [11,21,39]. Multivariate models that include ETS exposure and its related factors may be difficult to interpret due to potential collinearity. Smoking status has a well-known association with socioeconomic status (SES) [41], and SES is predictive of ETS exposure in children [42]. Therefore, SES may be a proxy for global ETS exposure over the early years of life. In addition to SES, smoking status is also predicted by race, educational attainment, and marital status [43]. Few of the multivariate studies provided a rationale for their selection of control variables. In reporting of future studies, greater details about the multivariate modeling steps may aid in assessment of collinearity when significant bivariate outcomes become nonsignificant in multivariate analysis.

This review has several limitations. The search was limited to studies published from 1990 to April 2009 in the English language. We searched only Pubmed and Embase and did not attempt to locate unpublished studies. The nature of the primary studies precluded a meta-analysis. The large retrospective database analyses included in this study (Table 2) all depended on bronchiolitis or RSV disease classification from diagnostic codes or medical record diagnosis. Although RSV is a leading cause of LRTI in infants and children, identifying the etiology of LRTI is not systematically undertaken in EDs or physician offices. Methods and reporting of ETS exposure ascertainment in these studies also varies widely. However, in most studies, data on the child’s ETS exposure level ultimately are gathered from parent or caregiver report, whether through direct data collection for the study or, for example, through retrospective review of the mother’s prenatal health records. Our review highlights the inherent difficulty of accurately assessing global ETS exposure.

## Conclusion

Overall we found ample evidence that ETS exposure places infants and young children at increased risk of hospitalization for RSV-attributable LRTIs, and increases the severity of illness as measured by degree of hypoxia among children hospitalized for RSV. Based on a small number of studies, we also found evidence that ETS exposure does not increase the likelihood among general populations with RSV of hospitalization versus outpatient treatment.

The prevention of serious RSV illness provides one more rationale for protecting infants and young children from exposure to tobacco smoke, especially high-risk groups such as premature infants and those with chronic conditions who are considered at increased risk of serious RSV disease.

## Competing interests

This project was funded under a contract with MedImmune, LLC. Joseph R. DiFranza, MD, provided consultant services to MedImmune. Amy M. Barrett, MSPH, MA, and Ann D. Colosia, PhD, are employees of RTI Health Solutions and provided consulting services to MedImmune. Parthiv J. Mahadevia, MD, MPH, and Anthony Masaquel, PhD, MPH, are employees of MedImmune. All authors agreed on the final text and conclusions of the manuscript. There are no other competing interest disclosures.

## Authors’ contributions

JD participated in the conception of the literature review and paper, reviewed published studies, analyzed data, and drafted portions of the manuscript. AM participated in the conception of the literature review and paper, reviewed studies, analyzed data, and critically revised the manuscript for important intellectual content. AB and AC each participated in the design and conception of the literature review and paper, reviewed published studies, extracted and analyzed data, and drafted portions of the manuscript. PM participated in the conception of the manuscript, analyzed data, and critically revised the manuscript for important intellectual content. All authors read and approved the final manuscript.

## Pre-publication history

The pre-publication history for this paper can be accessed here:

http://www.biomedcentral.com/1471-2431/12/81/prepub

## Supplementary Material

Additional file 1 Table S1. Provides full bias assessment of studies reported in this systematic literature review. Click here for file

Additional file 2 ** PRISMA Checklist.** Provides location in manuscript where PRISMA topics are reported. Click here for file
