# Supplementary material for: Systematic literature review assessing tobacco smoke exposure as a risk factor for serious respiratory syncytial virus disease among infants and young children
Source: BMC Pediatr. 2012 Jun 21;12:81. doi: 10.1186/1471-2431-12-81 (PMC3411420; doi:10.1186/1471-2431-12-81)
Supplement: Additional file 1 — Table S1. Provides full bias assessment of studies reported in this systematic literature review. [file 1471-2431-12-81-S1.pdf]

Risk of Bias Related to Assessing the Association of Serious RSV and Environmental Tobacco Smoke (ETS) Exposure[a]

		Disease misclassification: Was RSV diagnosis confirmed by laboratory testing?		Free of selective reporting?		Free of other bias related to enviornmental tobacco smoke (ETS) exposure or outcomes assessment?	
**Study type**	**Reference**	**Judgment**	**Description**	**Judgment**	**Description**	**Judgment**	**Description**
**Case-control**	Al-Shehri MA, Sadeq A, Quli K: Bronchiolitis in Abha, Southwest Saudi Arabia: viral etiology and predictors for hospital admission. West Afr J Med 2005, 24(4):299-304.	Unclear	Cases only were tested for respiratory viruses with enzyme immunoassay and fluorescent antibody tests. 40% were RSV-positive	Low risk	Multivariate analyses fully reported	High risk	Both cases and controls were diagnosed with bronchiolitis (participant selection bias)
**Cohort, cross-sectional**	Al-Sonboli N, Hart CA, Al-Aghbari N, Al-Ansi A, Ashoor O, Cuevas LE: Human metapneumovirus and respiratory syncytial virus disease in children, Yemen. Emerg Infect Dis 2006, 12(9):1437-1439.	Unclear	Children with acute respiratory infections were recruited and tested for specific viruses; 44% were RSV-positive	Low risk	Multivariate analyses fully reported	Unclear	No report of how smoke exposure or other family characteristics were ascertained (exposure bias)
**Cohort**	Boyce TG, Mellen BG, Mitchel EF, Jr., Wright PF, Griffin MR: Rates of hospitalization for respiratory syncytial virus infection among children in medicaid. J Pediatr 2000, 137(6):865-870.	High risk; could underestimate ETS effect	ICD-9 codes for RSV infection or bronchiolitis at hospitalization	Low risk	Multivariate analyses fully reported	Low risk	No other bias concerns related to ETS exposure or outcomes assessment
**Cohort, cross-sectional**	Bradley JP, Bacharier LB, Bonfiglio J, Schechtman KB, Strunk R, Storch G, Castro M: Severity of respiratory syncytial virus bronchiolitis is affected by cigarette smoke exposure and atopy. Pediatrics 2005, 115(1):e7-14.	Low risk	Lab-confirmed RSV: RSV enzyme immunoassay, direct fluorescent antibody assay, or viral culture	Low risk	Multivariate analyses fully reported	Low risk	No other bias concerns related to ETS exposure or outcomes assessment
**Cohort**	Broughton S, Roberts A, Fox G, Pollina E, Zuckerman M, Chaudhry S, Greenough A: Prospective study of healthcare utilisation and respiratory morbidity due to RSV infection in prematurely born infants. Thorax 2005, 60(12):1039-1044.	Low risk	Lab-confirmed RSV from nasopharyngeal aspirates using immunofluorescence and culture for RSV	Low risk	Multivariate analyses fully reported	Low risk	No other bias concerns related to ETS exposure or outcomes assessment
**Case-control**	Bulkow LR, Singleton RJ, Karron RA, et al. Risk factors for severe respiratory syncytial virus infection among Alaska native children. Pediatrics 2002; 109:210-6.	Low risk	Lab-confirmed RSV with enzyme immunoassay and fluorescent antibody tests; patients only had to be positive by one test, but were doubly positive in 81%.	Low risk	Multivariate analyses fully reported	Unclear	Authors noted the potential for misclassification of ETS exposure because Alaska native children spend much time indoors during the winter RSV season and could be exposed to ETS when visiting other houses or community buildings.
**Cohort**	Carbonell-Estrany X, Quero J: Hospitalization rates for respiratory syncytial virus infection in premature infants born during two consecutive seasons. Pediatr Infect Dis J 2001, 20(9):874-879.	Low risk	Lab-confirmed RSV by rapid antigen testing (ELISA or immunofluorescence) in 97% of the 75.4% of rehospitalized children tested; viral cultures in the other 3%.	Low risk	Multivariate analyses fully reported	Low risk	No other bias concerns related to ETS exposure or outcomes assessment
**Cohort**	Carroll KN, Gebretsadik T, Griffin MR, Dupont WD, Mitchel EF, Wu P, Enriquez R, Hartert TV: Maternal asthma and maternal smoking are associated with increased risk of bronchiolitis during infancy. Pediatrics 2007, 119(6):1104-1112.	High risk; could underestimate ETS effect	ICD-9 codes for RSV infection or bronchiolitis and RSV pneumonia at hospitalization	Low risk	Multivariate analyses fully reported	Low risk	No other bias concerns related to ETS exposure or outcomes assessment
**Cohort**	Chatzimichael A, Tsalkidis A, Cassimos D, Gardikis S, Tripsianis G, Deftereos S, Ktenidou-Kartali S, Tsanakas I: The role of breastfeeding and passive smoking on the development of severe bronchiolitis in infants. Minerva Pediatr 2007, 59(3):199-206.	Unclear	Clinical diagnosis: study participants were infants hospitalized for acute bronchiolitis	Low risk	Multivariate analyses fully reported	Low risk	No other bias concerns related to ETS exposure or outcomes assessment

Risk of Bias Related to Assessing the Association of Serious RSV and Environmental Tobacco Smoke (ETS) Exposure[a]

		Disease misclassification: Was RSV diagnosis confirmed by laboratory testing?		Free of selective reporting?		Free of other bias related to enviornmental tobacco smoke (ETS) exposure or outcomes assessment?	
**Study type**	**Reference**	**Judgment**	**Description**	**Judgment**	**Description**	**Judgment**	**Description**
**Case-control**	Figueras-Aloy J, Carbonell-Estrany X, Quero J: Case-control study of the risk factors linked to respiratory syncytial virus infection requiring hospitalization in premature infants born at a gestational age of 33-35 weeks in Spain. Pediatr Infect Dis J 2004, 23(9):815-820.	Low risk	Lab-confirmed RSV by immunofluorescence assay, ELISA, or virus culture.	Low risk	Multivariate analyses fully reported	Low risk	No other bias concerns related to ETS exposure or outcomes assessment
**Cohort (2-cohort)**	Figueras-Aloy J, Carbonell-Estrany X, Quero-Jimenez J, Fernandez-Colomer B, Guzman-Cabanas J, Echaniz-Urcelay I, Domenech-Martinez E: FLIP-2 Study: risk factors linked to respiratory syncytial virus infection requiring hospitalization in premature infants born in Spain at a gestational age of 32 to 35 weeks. Pediatr Infect Dis J 2008, 27(9):788-793.	Low risk	Lab-confirmed RSV by immunofluorescence assay, ELISA, or virus culture.	Low risk	Multivariate analyses fully reported	Low risk	No other bias concerns related to ETS exposure or outcomes assessment
**Cohort**	Gavin NI: Predictive accuracy of risk factors for RSV-related hospitalizations among infants in low-income families born at 32 to 35 weeks of gestation. J Clin Outcomes Manage 2007, 14(6):323-331.	High risk; could underestimate ETS effect	ICD-9-CM diagnostic codes and pathogen-unspecified codes for acute bronchiolitis, viral pneumonia, bronchopneumonia, and pneumonia used	Low risk	Multivariate analyses fully reported	Low risk	No other bias concerns related to ETS exposure or outcomes assessment
**Cohort**	Groothuis JR, Gutierrez KM, Lauer BA: Respiratory syncytial virus infection in children with bronchopulmonary dysplasia. Pediatrics 1988, 82(2):199-203.	Low risk	Lab-confirmed RSV by rapid RSV antigen tests (fluorescent antibody test and enzyme immunoassay) and for virus culture; RSV developed in 59%	Low risk	Bivariate analysis fully reported	High risk	No multivariate analysis performed (confounding bias)
**Case-control**	Gurkan F, Kiral A, Dagli E, Karakoc F: The effect of passive smoking on the development of respiratory syncytial virus bronchiolitis. Eur J Epidemiol 2000, 16(5):465-468.	Low risk	Lab-confirmed RSV by viral antigen isolation of RSV from nasopharyngeal washes	Low risk	Bivariate analysis fully reported	High risk	No multivariate analysis performed (confounding bias)
**Case-control**	Hall CB, Hall WJ, Gala CL, MaGill FB, Leddy JP: Long-term prospective study in children after respiratory syncytial virus infection. J Pediatr 1984, 105(3):358-364.	Low risk	Lab-confirmed RSV by viral culture	Low risk	Long-term study with results fully described	High risk	No multivariate analysis performed (confounding bias)
**Prospective surveillance**	Hall CB, Weinberg GA, Iwane MK, Blumkin AK, Edwards KM, Staat MA, Auinger P, Griffin MR, Poehling KA, Erdman D et al: The burden of respiratory syncytial virus infection in young children. N Engl J Med 2009, 360(6):588-598.	Low risk	Lab-confirmed RSV by culture and reverse-transcriptase polymerase chain reaction (18% of the 5067 specimens were RSV-positive, and analysis was performed on this subgroup)	Low risk	Multivariate analyses fully reported	Low risk	No other bias concerns related to ETS exposure or outcomes assessment
**Case-control**	Hayes EB, Hurwitz ES, Schonberger LB, Anderson LJ: Respiratory syncytial virus outbreak on American Samoa. Evaluation of risk factors. Am J Dis Child 1989, 143(3):316-321.	Unclear	Most patients had clinical bronchiolitis; 34 of 35 patients tested, 53% were lab-confirmed RSV-positive	Low risk	Risk factors and related outcomes reported for cases, well controls, and ill controls	High risk	No multivariate analysis performed (confounding bias)
**Cohort**	Holberg CJ, Wright AL, Martinez FD, Ray CG, Taussig LM, Lebowitz MD: Risk factors for respiratory syncytial virus-associated lower respiratory illnesses in the first year of life. Am J Epidemiol 1991, 133(11):1135-1151.	Low risk	Lab-confirmed RSV by viral culture and immunofluorescent techniques. An episode was considered to be RSV positive if either culture, immunofluorescence, or both were positive.	Low risk	Multivariate analyses fully reported	Low risk	No other bias concerns related to ETS exposure or outcomes assessment

Risk of Bias Related to Assessing the Association of Serious RSV and Environmental Tobacco Smoke (ETS) Exposure[a]

		Disease misclassification: Was RSV diagnosis confirmed by laboratory testing?		Free of selective reporting?		Free of other bias related to enviornmental tobacco smoke (ETS) exposure or outcomes assessment?	
**Study type**	**Reference**	**Judgment**	**Description**	**Judgment**	**Description**	**Judgment**	**Description**
**Case-control**	Holman RC, Shay DK, Curns AT, Lingappa JR, Anderson LJ: Risk factors for bronchiolitis-associated deaths among infants in the United States. Pediatr Infect Dis J 2003, 22(6):483-490.	High risk	Not lab-confirmed RSV; bronchiolitis code on infant death certificate	Low risk	Multivariate analyses fully reported	Low risk	No other bias concerns related to ETS exposure or outcomes assessment
**Cohort**	Koehoorn M, Karr CJ, Demers PA, Lencar C, Tamburic L, Brauer M: Descriptive epidemiological features of bronchiolitis in a population-based cohort. Pediatrics 2008, 122(6):1196-1203.	High risk; could underestimate ETS effect	Not lab-confirmed RSV; bronchiolitis cases identified from ICD-9 diagnosis codes for acute bronchitis and bronchiolitis in outpatient medical charts or acute bronchiolitis in hospital discharge records	Low risk	Multivariate analyses fully reported	Low risk	No other bias concerns related to ETS exposure or outcomes assessment
**Cohort**	Law BJ, Langley JM, Allen U, et al. The Pediatric Investigators Collaborative Network on Infections in Canada study of predictors of hospitalization for respiratory syncytial virus infection for infants born at 33 through 35 completed weeks of gestation. Pediatr Infect Dis J 2004; 23:806-14.	Low risk	Lab-confirmed RSV by viral culture and/or rapid test.	Low risk	Multivariate analyses fully reported	Low risk	No other bias concerns related to ETS exposure or outcomes assessment
**Cohort**	Marbury MC, Maldonado G, Waller L: The indoor air and children's health study: methods and incidence rates. Epidemiology 1996, 7(2):166-174.	High risk; could underestimate ETS effect	Diagnosis of lower-respiratory illness and bronchiolitis obtained from electronic medical records	Low risk	Multivariate analyses fully reported	Low risk	No other bias concerns related to ETS exposure or outcomes assessment
**Case-control**	McConnochie KM, Roghmann KJ: Parental smoking, presence of older siblings, and family history of asthma increase risk of bronchiolitis. Am J Dis Child 1986, 140(8):806-812.	High risk; could underestimate ETS effect	Diagnosis of bronchiolitis recorded in the outpatient medical record	Low risk	Multivariate analyses fully reported	Low risk	No other bias concerns related to ETS exposure or outcomes assessment
**Case-control**	Nielsen HE, Siersma V, Andersen S, et al. Respiratory syncytial virus infection--risk factors for hospital admission: a case-control study. Acta Paediatr 2003;92:1314-21.	Low risk	Lab-confirmed RSV by direct immunofluorescence antigen testing of nasopharyngeal aspirates	Low risk	Multivariate analyses fully reported	Low risk	No other bias concerns related to ETS exposure or outcomes assessment
**Cohort**	Reese AC, James IR, Landau LI, Lesouef PN: Relationship between urinary cotinine level and diagnosis in children admitted to hospital. Am Rev Respir Dis 1992, 146(1):66-70.	High risk; could underestimate ETS effect	Participants were children admitted to hospital with a variety of diagnoses (including respiratory illness) for whom a urinary cotinine level was available; they were given a respiratory illness questionnaire	High risk	"Multivariate statistical analysis" carried out, but results not clearly reported. No detail was provided on which factors were included in the model or the dependent variable of the model	Unclear	Risk of confounding not clear; regression analysis appears to have been performed but was insufficiently reported so not possible to tell which factors were controlled for
**Case-control**	Reeve CA, Whitehall JS, Buettner PG, et al. Predicting respiratory syncytial virus hospitalisation in Australian children. J Paediatr Child Health 2006;42:248-52.	Low risk	Lab-confirmed RSV by direct immunofluorescence testing of nasopharyngeal aspirates	Low risk	Multivariate analyses fully reported	Low risk	No other bias concerns related to ETS exposure or outcomes assessment
**Cohort, cross-sectional**	Somech R, Tal G, Gilad E, Mandelberg A, Tal A, Dalal I: Epidemiologic, socioeconomic, and clinical factors associated with severity of respiratory syncytial virus infection in previously healthy infants. Clin Pediatr (Phila) 2006, 45(7):621-627.	Low risk	Lab-confirmed RSV by enzyme immunoassay	Low risk	Analysis fully reported	High risk	No multivariate analysis performed (confounding bias)

Risk of Bias Related to Assessing the Association of Serious RSV and Environmental Tobacco Smoke (ETS) Exposure[a]

		Disease misclassification: Was RSV diagnosis confirmed by laboratory testing?		Free of selective reporting?		Free of other bias related to enviornmental tobacco smoke (ETS) exposure or outcomes assessment?	
**Study type**	**Reference**	**Judgment**	**Description**	**Judgment**	**Description**	**Judgment**	**Description**
**Case-control**	Stensballe LG, Kristensen K, Simoes EA, Jensen H, Nielsen J, Benn CS, Aaby P: Atopic disposition, wheezing, and subsequent respiratory syncytial virus hospitalization in Danish children younger than 18 months: a nested case-control study. Pediatrics 2006, 118(5):e1360-1368.	Low risk	Lab-confirmed RSV through database containing "result of the RSV test"	Low risk	Multivariate analyses fully reported	Low risk	No other bias concerns related to ETS exposure or outcomes assessment
**Cohort, cross-sectional**	Sritippayawan S, Prapphal N, Wong P, Tosukhowong P, Samransamruajkit R, Deerojanawong J: Environmental tobacco smoke exposure and respiratory syncytial virus infection in young children hospitalized with acute lower respiratory tract infection. J Med Assoc Thai 2006, 89(12):2097-2103.	Low risk	Children hospitalized for LRI were included; lab-confirmed RSV (RSV antigen assay performed by indirect immunofluorescent antibody technique) group compared to non-RSV group; urinary cotinine measured and technician blinded to patient data	High risk	"Multinomial logistic regression" performed, but results for only one significant factor were reported, and other factors included in model were not provided	Unclear	Risk of confounding not clear; regression analysis appears to have been performed but was insufficiently reported so not possible to tell which factors were controlled for
**Cohort**	von Linstow ML, Hogh M, Nordbo SA, Eugen-Olsen J, Koch A, Hogh B: A community study of clinical traits and risk factors for human metapneumovirus and respiratory syncytial virus infection during the first year of life. Eur J Pediatr 2008, 167(10):1125-1133.	Low risk	Lab-confirmed RSV from nasal swabs of symptomatic children	Low risk	Multivariate analyses fully reported	Low risk	No other bias concerns related to ETS exposure or outcomes assessment

[a] Risk of bias related to adequate sequence generation, allocation concealment, blinding of participants and personnel, or blinding of outcome assessment is not relevant to noninterventional studies such as the ones included in this literature review. Assessments for these types of bias have not been included as all would be "not applicable."

Description of Ratings for Assessment of Bias Risk Related to the Association of Serious RSV and Environmental Tobacco Smoke Exposure

	Low Risk	High Risk	Unclear
**Disease misclassification**	Lab-confirmed RSV	Diagnostic codes or diagnoses recorded from medical records (various) or cause of death from death certificate (bronchiolitis)	Clinical diagnosis or analysis performed on data from participants with a variety of lower respiratory tract infections
**Selective reporting**	Variables assessed in models or analyzed are adequately described and significant results are detailed	Study results not adequately or clearly reported (e.g., list of factors included in the multivariate model not provided)	[None]
**Other bias**			
Confounding bias	Multivariate analysis	Univariate or bivariate analysis only	Inadequate reporting makes it impossible to tell which potentially confounding factors were controlled for
Exposure bias	Passive smoke exposure assessed through cotinine level, parent-reported questionnaire, maternal smoking data from birth certificate, or medical records	[None]	Smoke exposure variable not defined
Participant selection bias in case-control studies	Healthy controls without RSV or respiratory disease	Controls were ill with non-RSV disease (instead of healthy controls)	Not possible to determine health status of controls
